# Evaluating functional ability in older adults’ object retrieval behavior from kitchen furniture using OpenPose and REBA

**DOI:** 10.1038/s41598-024-75470-6

**Published:** 2024-10-26

**Authors:** Chengmin Zhou, Ruolan Yu, Jake Kaner

**Affiliations:** 1 College of Furnishings and Industrial Design, Nanjing Forestry University, Nanjing, 210037 Jiangsu China; 2 Jiangsu Co-Innovation Center of Efficient Processing and Utilization of Forest Resources, Nanjing, 210037 Jiangsu China; 3 School of Art and Design, Nottingham Trent University, Nottingham, NG1 4FQ UK

**Keywords:** The elderly, Openpose technology, REBA assessment method, Behavioural ability to pick up objects, Aging-friendly design of cabinets, Ageing, Geriatrics, Health policy, Quality of life, Human behaviour

## Abstract

The purpose of this study is to evaluate, through analysis, the ability of older persons to retrieve items from kitchen cabinets. To achieve this goal, data were collected from 128 valid questionnaires and supplemented with field research and user interviews. The study revealed that the elderly’s behavior in retrieving items from kitchen spaces is characterized by both high frequency and difficulty. For this experiment, a total of 42 participants, comprising 21 males and 21 females from the self-care elderly population in the Yangtze River Delta region, were recruited. Two different experimental settings were arranged: one with kitchen utensils arranged in a straight line and another with a purpose-made chest of drawers with varying heights. Video recordings using the Logitech C930C were utilized to capture the gestures and behaviors of the elderly while retrieving objects from the kitchen cupboards (cabinets). By employing a combination of the OpenPose model and the Rapid Entire Body Assessment (REBA) method, which involves calculating human posture angles, REBA scores, and determining the risk level of Work-Related Musculoskeletal Disorders (WMSDs), a risk assessment framework for manual operations associated with WMSDs was developed. Using the angle data acquired from the user operation experiment as parameters, a gradient model of the elderly user’s operational capability was established. The findings indicated a significant impact of neck, trunk, and knee movements on the subjects (*P* < 0.001). The participants were able to distinguish between different levels of exertion, categorizing movements as ‘easy’, ‘moderate,’ or ‘strenuous.’ These results form the basis for a comfort gradient model for leaning over and retrieving items. Given the prevalent conditions of bone and joint degeneration and osteoporosis among the elderly population, it is evident that they face challenges when accessing items in the kitchen. Therefore, investigating the elderly’s execution abilities during the retrieval process becomes crucial. Understanding how different cabinet heights impact the joint angles of the elderly can be instrumental in optimizing cabinet designs for elderly users, thereby reducing their physical exertion in the kitchen and enhancing their comfort levels. This research holds significant value in improving the quality of life for the elderly population at home and fostering the advancement of elderly-friendly design principles.

According to the standards of the World Health Organization, countries with a population over 60 years old of more than 10% or a population over 65 years old of more than 7% are called ‘aging countries’^1^. In the twenty-first century, China is the most populous country in the world, which has fully enjoyed the economic growth brought by the “demographic dividend” in the past 30 years, and has quickly entered an aging society, with the largest elderly population in the world^2^. Around 2035, the number of elderly people aged 60 and above will exceed 400 million, accounting for more than 30% of the total population, and will enter the stage of severe aging^3,4^. The arrival of an aging society is an urgent problem, which will affect everyone’s life. With this background, the industry of knowledge, health and care for the aged has become a new growth point under the dual-cycle situation in China. With the help of scientific research and emerging technologies, it has become a research hotspot, an industrial trend at present and a requirement for the future to develop healthy and comfortable home environment such as furniture products for the elderly. With the development of society, the concepts of smart home technology have become more and more popular^5,6^. In recent years, the rapid growth of IOT (Internet of things) components and devices has promoted the expansion of IOT solutions based on smart home technology^7,8^. Among them, the elderly-oriented smart home has attracted increasing attention^9^. However, not all elderly people can accept the smart home^10^, as many old people are reluctant to use new technologies. Many products are designed in a way that does not consider the emotional preferences and spiritual demands of the elderly. For various reasons, many elderly are not willing to use smart home products^11^. Therefore, in the design of smart home products, it is particularly important to pay attention to users’ emotional needs and interactive experience. With the deepening of social aging in China, according to the data of the Ministry of Civil Affairs, the proportion of empty nesters among the elderly population in China is now more than half, and in some big cities and rural areas, the proportion of empty nesters even exceeds 70%^12^. According to the survey, the elderly in China prefer home-based care for the aged^13^, but the current home facilities do not take the elderly as the main analysis users.

The kitchen is an important activity place in home life, being the most frequently used place in the daily life of the elderly^14^. As the most important facility in the kitchen space, the kitchen cabinet has the closest contact with the human body and the highest frequency of use, and its design has the most direct impact on the safety and comfort of users’ operation^15^. However, when the kitchen is operated for too long or too frequently, it is easy to cause problems, such as muscle soreness and joint stiffness for older persons, which will lead to serious capacity loss and even dangerous accidents^16,17^. Therefore, it is important to ensure the safety, convenience and comfort of the elderly in the kitchen, which can improve their operating efficiency. The design of geriatric designed kitchen cabinets is based on the principle of ergonomics, starting from the layout, shape, size and function, so as to create convenient and practical kitchen cabinets that are safe and enable a healthy living environment to improve quality of life^18^. However, at present, the design of kitchen cabinets is mostly standardised, and the target group is mainly young and middle-aged people, with little consideration given to the special needs of older generation. Therefore, under the background of an aging population and elderly independence as the research object, kitchen behaviour is deeply studied to identify the operational needs and improve the kitchen experience of the older person^19^.

Research on kitchen aging can be roughly divided into three levels: kitchen system; user ability; and influencing factors. First of all, on the research level of kitchen system, Câmara et al.^20^ put forward the difficulties and needs of the elderly in using the kitchen from the perspective of ergonomics, and provided suggestions for improvement. Asghar et al.^21^, attempted to understand the cognitive impairment of the elderly, a kitchen-assisted search system consisting of a projector, a camera and RFID (Radio Frequency Identification) was set up. The system uses sensing and projection technology to find the position of the desired object and guide the elderly to successfully find the desired object placed in the kitchen. Yared et al.^22^, designed and developed a cooking safety system consisting of sensor nodes, microcontrollers and computing units for the physiological decline and memory impairment of the elderly. At the same time, an inference engine system based on risk prevention algorithm and fuzzy logic technology is constructed to prevent three kinds of risks (fire, burn and gas/smoke poisoning) in cooking.

On the research level of users’ ability, Robert et al.^23^, designed the kitchen cabinet based on questionnaires and interviews, in order to solve dangerous situations, such as burns and cuts, hitting the door, and avoiding sharp edges or protruding handles of the kitchen cabinet. Ficocelli et al.^24^, focused on the cognitive impairment of the elderly, designed and developed an auxiliary kitchen system, consisting of a user interface and an automatic cabinet to help the elderly with cognitive impairment to complete the daily living ability test, such as retrieving and storing items and obtaining recipes for meal preparation. Yuan et al.^25^, designed and developed an automatic temperature monitoring system at the top of the stove to improve the safety of the kitchen. Zubaidi et al.^26^, investigated the barriers of elderly users with arthritis. The QFD (Quality Function Deployment) method was used to determine the needs of consumers, and an ergonomic chopping block kit consisting of a knife and a chopping block was designed and developed. Wang et al.^27^, asked the elderly in the non-cognitive impairment group and the cognitive impairment group to complete three kitchen tasks respectively, so as to explore the relationship between the efficiency of daily activities of the elderly and the spatial layout of the kitchen, and evaluate the regulatory role of cognitive function.

On the research level of influencing factors, Kelsheimer et al.^28^ investigated the frequency and difficulty of using kitchen items to explore which operations have obstacles and obtain the actual effect after using assistive devices. The research of Hrovatin et al.^29^ shows that the elderly have the following problems when using the kitchen: insufficient lighting, improper combination of working faces, difficulty in sanitary care, improper shape of furniture and tasks that become troublesome due to memory loss. Schulz et al.^30^ pointed out that people’s willingness to purchase technical assistance at their own expense, such as kitchen work and personal care, depends on income, privacy issues, perceived future needs and possible race and ethnicity. Kang et al.^31^ put forward a universal kitchen design for the elderly and the disabled. Based on the research on the mobility of wheelchair users, five analysis criteria are formulated: floor space, workflow, universal accessibility, subsequent use area and safety.

In the existing research, there are many methods to evaluate the human load state^32,33^. The method of subjective questionnaire fatigue investigation is to let the subjects describe their subjective fatigue feelings by questionnaire survey, and provide a variety of information about human fatigue, such as the time of fatigue, the location of fatigue, the degree of fatigue and so on. The survey methods of subjective questionnaire often need to be combined with field observation, interview and objective experiment to analyze each other. Beata Fabissiak’s^34^ team used a Likert scale questionnaire survey and direct interviews to ask the elderly of different ages to rate the difficulty of different kitchen activities. The results suggest that the design of standing kitchen cabinets should be adopted as much as possible for elderly users. Ibrahim and Davies^35^, also used questionnaires to study the difficulty of kitchen behaviour, and found that cooking difficulties were caused by unreasonable kitchen design and the decline of functional ability of the elderly. Jasna Hrovatin et al.^29^ conducted a survey on the satisfaction of the elderly in using kitchen functions through household surveys. The results show that the main problems faced by elderly users when using the kitchen include: the task becomes troublesome due to memory decline, the furniture is not suitable in shape and size, and it is inconvenient to clean.

Objective experimental fatigue measurement refers to a research method that uses physiological measuring instruments to present the whole process in the form of data to provide objective reference for various physiological indexes of the human body, and at the same time, it is often combined with a subjective questionnaire survey to provide more accurate and reasonable fatigue evaluation guidance^36^. Jun Ji and Hong Jin^37^, detected the changes of heart rate during four different kitchen tasks, and found that the intensity of physical exertion of human body was basically positively correlated with the heart rate, thus the degree of physical fatigue of human body could be detected. However, the accuracy of this method has yet to be confirmed. Therefore, after comparison, researchers choose to detect the changes of surface EMG signals as the research method of human local fatigue. Currently, surface electromyography technology has been widely used in the detection and analysis of human fatigue. Shankar Subramaniam^38^ and other researchers collected the EMG of the left and right trapezius muscles and erector spinae’s surface of human body performing four kinds of kitchen tasks, and combined with the subjective questionnaire survey and analysis, put forward that rolling and baking tasks that would lead to the increasing fatigue of workers’ shoulders and waist muscles within 30 minutes. Fong-Gong Wu^39^ compared the differences before and after designing an auxiliary wearing system for cooking hands in the kitchen of the elderly by collecting and analyzing electromyography (EMG) data. It was found that the difference before and after wearing the muscle system is significant, and the decrease of surface EMG parameters proves that the auxiliary system can reduce the fatigue of hand muscles. It shows that the method of surface electromyography plays a guiding role in the process of detecting and reducing fatigue in the design of kitchen related products. Surface electromyography (SEMG) evaluation has the characteristics of sensitivity, accuracy and objectivity, and there is a good consistency between the changes of EMG signals and the state of muscle activity, so SEMG is an effective method to study human muscle fatigue under kitchen work.

Numerous studies and techniques use or develop computer vision techniques for observation-based ergonomics assessments. Computer vision techniques have become integral in the field of physical ergonomic assessments, particularly for evaluating repetitive motion tasks and postural risks. These techniques utilize advanced image processing and machine learning algorithms to analyze human motion and posture from video data, providing detailed insights into ergonomic risks and potential improvements. For instance, a study by Greene et al.^40^ demonstrated the use of computer vision to visualize stressful aspects of repetitive tasks, helping to identify specific job factors contributing to physical stress and suggesting ergonomic improvements through heat maps superimposed on video frames. Moreover, Fernández et al.^41^ developed a method that automatically computes Rapid Upper Limb Assessment (RULA) scores from digital video using computer vision and machine learning techniques. This method enhances the accuracy and efficiency of ergonomic risk assessments by detecting body-joint positions and angles, even under challenging conditions such as poor illumination or occlusions. Another application by Kunz et al.^42^ utilized AI-driven video-based approaches for 3D body posture analysis and repetition counting in advanced manufacturing settings. This method supports real-time ergonomic and fatigue analyses, enhancing the health and productivity of human operators by providing accurate tracking and assessment of joint motions (Kunz et al.^42^). Egeonu and Jia^43^ conducted a systematic literature review on computer vision-based biomechanical models. Their study demonstrates that computer vision-based biomechanical models have significant advantages in workload estimation, including low cost, non-invasiveness, applicability in both indoor and outdoor environments, and automated assessment capabilities. However, current computer vision-based biomechanical models also face challenges in workload estimation, primarily due to issues of occlusion and insufficient accuracy in posture monitoring. Particularly in complex work environments, there is a lack of benchmark datasets to evaluate the accuracy of these methods. Some anthropometric data, such as body weight and volume, require additional steps to obtain. These issues limit the system’s ability for automatic monitoring in dynamic environments. Although some studies have attempted to address these problems through methods like multi-view fusion, pose completion algorithms, and automatic calibration, these issues have not been fully resolved, affecting the precision and generalizability of the models in practical applications. These advancements in computer vision for ergonomic assessments enable more precise and efficient evaluations of physical workload, contributing to the development of safer and more comfortable work environments. By integrating these technologies, researchers and practitioners can better understand and mitigate the risks associated with various physical tasks, ultimately improving worker health and productivity.

When evaluating the working posture, the researcher makes a more detailed assessment of the posture fatigue and the risk of musculoskeletal diseases in the process of various behaviours by dividing the kitchen behaviours, and optimises them after discovering unreasonable working postures, so as to guide users to reduce the fatigue caused by kitchen activities and prevent the occurrence of chronic diseases. Relevant analysis methods of behaviour observation mainly include: subjective scale method, interview method, psychophysiological recording method, OWAS (Ovako Working Posture Analysis System)^44^, RULA (Rapid Upper Limb Assessment)^45^, REBA (Rapid Entire Body Assessment) and so on. What these methods have in common is that the working posture must be observed and evaluated. At present, the commonly used human posture and behaviour analysis tools mainly include: CAPTIV human behaviour analysis system in France, Mangold INTERACT behaviour analysis system in Germany, and Noldus The Observer XT behaviour observation and analysis system in the Netherlands. Jong-Yu Chyuan and Amit Bhatia^46,47^, used the method of RULA to study the working posture and potential risk factors of different kitchen workers. It is pointed out that there is a great posture load and risk in the back of kitchen workers, and the posture should be adjusted in time. Most of the data of posture evaluation methods are based on observation, or based on observation, the fatigue degree of human body is simulated and evaluated by modern human factor engineering software, rather than on experimental data to judge the fatigue degree of muscles in various postures. However, its advantage is that the method of objective experiment is simpler and faster, and it can be selected according to the needs of the scene. REBA is a sensitive tool for evaluating the abnormality of various parts of the body (wrist, upper arm, forearm, neck, trunk and leg)^48^. The REBA method was developed by Hignett et al.^49^, and is widely used in the assessment of musculoskeletal diseases in manufacturing, agriculture and other industries. Its basic principle is to detect six kinds of body joint angles (trunk, upper arm, lower limb, neck, lower arm and wrist) and three influencing factors (load, grip and activity frequency) in the work behaviour, and obtain corresponding scores by comparing with the scoring table, and accumulate them to get the total score, which represents the degree of harm of the work posture^50^.

As the issue of population aging becomes increasingly severe, ensuring the quality of life for elderly individuals has become particularly important. The kitchen, being a frequently used space in daily life, has a direct impact on the safety and comfort of the elderly. In the kitchen, elderly individuals often need to bend, reach, and retrieve items, which can lead to musculoskeletal injuries and increase the risk of accidents. Research has shown that improper kitchen design can result in musculoskeletal injuries for the elderly, making kitchen tasks more difficult and hazardous. Therefore, studying how to optimize kitchen design to reduce the physical burden and injury risk for elderly individuals is of significant importance. The primary objective of this study is to evaluate the ability of elderly individuals to retrieve and place items in the kitchen. Additionally, the study aims to assess the postural risks associated with these actions using the pose analysis tool (OpenPose) and the Rapid Entire Body Assessment (REBA) method. This study will first evaluate and analyse the behaviour of the elderly taking things from the cupboard through questionnaires, field research and user interviews. Then, combining OpenPose technology and REBA evaluation method, and taking the angle data generated in the user’s operation behaviour experiment as parameters, a gradient model of elderly users’ operation ability will be constructed. We will explore the influence of cabinet height on the actual joint angle of different parts of the elderly body and optimise the cabinet design of elderly users. With the results from this research, we aim to understand how to improve the comfort of the elderly in taking things in the kitchen and reduce the risk and burden of their operating behaviour^51^. The study found that postures adopted by elderly individuals during kitchen tasks significantly affect their physical discomfort (*P* < 0.001). Specifically, when retrieving items from high shelves, women exhibited significantly higher peak shoulder flexion angles compared to men, likely due to differences in height between genders. This finding highlights the impact of cabinet heights in kitchen design on the posture and discomfort of elderly individuals. For example, retrieving items from high cabinets requires significant shoulder and neck flexion, which can lead to muscle strain in these areas. Conversely, retrieving items from low cabinets involves frequent bending and kneeling, which increases the load on the lower back and knees. The cumulative effect of these postures can result in work-related musculoskeletal disorders (WMSDs) such as lower back pain, shoulder pain, and knee osteoarthritis. This study combines posture analysis and ergonomic assessment methods to reveal the impact of kitchen design on the operational behavior and physical discomfort of the elderly. Specifically, we propose an evaluation framework based on OpenPose and the Rapid Entire Body Assessment (REBA) method to analyze the postural load of elderly individuals during kitchen tasks. This framework provides a theoretical basis for optimizing kitchen design and reducing the operational burden on the elderly. Based on experimental data, we established comfort gradient models for the neck, torso, and knees, offering optimization recommendations for elderly individuals’ kitchen tasks. Our research not only contributes to improving the quality of life for the elderly but also promotes the development of age-friendly design principles, providing scientific evidence for the development of home products that better meet the needs of elderly individuals.

## Evaluation and analysis of behavioural ability of the elderly

### Behavioural needs analysis of the elderly

We first consolidated key findings from current research on age-friendly kitchen spaces and cabinetry elements. Although kitchen designs were not standardized across participants, we chose this approach to better reflect the diversity of real-world environments that elderly individuals operate in. By analyzing the impact of different kitchen layouts, we aimed to derive insights that can guide the design of more age-friendly kitchens. Different kitchen layouts, such as L-shaped, linear (one-wall), and U-shaped designs, can impact the usability and comfort for the elderly. For example, a linear (one-wall) layout may be more suitable for smaller kitchens, while U-shaped or island layouts can provide more workspace and storage in larger kitchens, thereby affecting operational efficiency and safety in kitchen activities. The L-shaped kitchen is particularly suitable for the elderly due to its smooth workflow and ample activity space. The storage area should center around the refrigerator, the washing area around the sink, and the cooking area around the stove, with distances between these zones ideally ranging from 4500 to 6000 mm. The minimum width for kitchen aisles should be 900 mm, with 1000 mm being generally preferable for safety and convenience. The width of countertops should be 550–600 mm, with an optimal height of 760 mm. Unloading spaces beside appliances should be approximately 390 mm wide, and placement spaces should be reserved on both sides of the sink and stove, with widths varying between 240 and 390 mm^52,53^. Understanding different kitchen layouts is crucial as it provides context for how elderly individuals navigate and interact with their kitchen environment. While this study primarily focuses on the physical capabilities of elderly participants in retrieving items from various cabinet heights, considering the diversity of kitchen designs allows for a more comprehensive understanding of their behavioral needs in real-world settings. Therefore, the discussion on kitchen layouts helps frame the environmental factors that could influence their operational behavior and inform future ergonomic design recommendations.

A total of 200 questionnaires were distributed in this survey, and a total of 128 valid questionnaires were recovered. We conducted a questionnaire survey on elderly kitchen behaviors from three aspects: basic information, usage experience, and expectations. This allowed us to understand the basic conditions of the elderly, gather their ratings on the difficulty of various kitchen tasks, identify causes of physical discomfort, and obtain their expectations for kitchen design. The survey was conducted through a combination of offline recruitment and online dissemination. Detailed questionnaire information can be found in Appendix A. In this survey, 52 elderly people aged 60–64 were surveyed, accounting for 40.63% of the total survey population. There were 26 elderly people aged 64–69, accounting for 20.31% of the total survey population. A total of 20 elderly people aged 70–74 accounted for 16.13% of the total survey population; 14 elderly people aged 75–79 accounted for 11.29% of the total survey population; 12 elderly people aged 80 or above, accounting for 9.68% of the total survey population. Since the survey requires the respondents to have many years of cooking experience in the kitchen, most of the respondents are women, accounting for 66.13% of the total number. Among the kitchen types surveyed, closed kitchens accounted for 67.74%, which was significantly higher than open kitchens (24.19%) and semi-open kitchens (8.06%). Regarding kitchen layouts, L-shaped layouts were the most common at 58.06%, followed by U-shaped layouts (29.03%) and linear (one-wall) layouts (11.29%).

In the survey, the section on the difficulty of kitchen tasks includes six scale analysis questions, composed of two dimensions. These questions use a seven-point Likert scale with the following levels: very easy, somewhat easy, slightly easy, neutral, slightly difficult, somewhat difficult, and very difficult. Participants rated the difficulty of various kitchen tasks based on their actual experiences. A score of 1 represents very easy, while a score of 7 represents very difficult.Combing and analysing the results of the questionnaire survey, it can be found that:

1. For the elderly, the difficulty of various kitchen tasks is ranked as follows: retrieving items from upper cabinets > retrieving items from lower cabinets > stir-frying food > washing dishes > cutting vegetables > washing vegetables. The specific difficulty scores are shown in Table 1. This indicates that completing storage tasks is more challenging compared to daily activities such as washing, cutting, cooking, and scrubbing. The difficulties arise from four main aspects: difficulty in squatting to retrieve items from lower cabinets, limited storage space, inconvenience in waste disposal, and the challenge of reaching items in upper cabinets due to height constraints. Moreover, retrieving and placing items in upper cabinets is more difficult than doing so in lower cabinets because the cabinet structure obstructs the elderly’s line of sight, making it hard for them to accurately select or place the required items.
2. When the elderly performs kitchen tasks, the waist and legs are the most common discomfort sites. Then, the correlation analysis was carried out on the difficulty of various behaviours in the kitchen and the discomfort of body parts, and the results are shown in Table 2. The *p*-values between the difficulty of picking up the items on the cabinet and the neck, shoulders, waist, arms, wrists, and legs are all greater than 0.05, and there is no significance. There is a significant relationship between the difficulty of taking items from the base cabinet and the discomfort of the waist and legs, and the correlation coefficient values are all greater than 0. Therefore, there is a positive correlation between the difficulty of taking items from the base cabinet and the discomfort of the waist and legs.
3. A variance analysis was performed on the difficulty of picking and placing items from the floor cabinets and the age of the survey respondents, and the results are shown in Table 3. It can be seen from the table that the difficulty of picking and placing items in the floor cabinet is as follows: the elderly over 80 years old > the elderly 75–79 years old > the elderly 70–74 years old > the elderly 65–69 years old > the elderly 60–64 years old. This result shows that for the elderly over 60 years old, the difficulty of picking and placing items in the base cabinet gradually increases with age. This may be because taking and placing items in the floor cabinet requires the mobilisation of the whole body’s bones to complete a series of actions such as bending over, bending legs, and reaching out to pick up items.

**Table 1:** Difficulty of kitchen tasks.

Kitchen tasks	Wash vegetables	Cut vegetables	Fried vegetables	Scrub	Pick up items from the cabinet	Get items from the base cabinet
Difficulty score	2.58	2.68	2.82	2.79	3.61	3.26

**Table 2:** Relationship between kitchen task difficulty and body uncomfortable parts.

	Neck	Shoulder	Waist	Arm	Wrists	Legs
Difficulty (wall cabinet)	0.191	0.048	0.031	− 0.008	− 0.014	0.088
Difficulty (under cabinet)	0.138	− 0.011	0.281*	0.112	0.105	0.311*

* p < 0.05.

**Table 3:** Difficulty in taking and placing items in the floor cabinet at different ages.

Age	60–64 (n = 52)	65–69 (n = 26)	70–74 (n = 20)	75–79 (n = 14)	> = 80 (n = 12)
Difficulty	2.93 ± 1.27	3.09 ± 1.30	3.33 ± 1.41	3.50 ± 1.52	4.17 ± 1.17

Based on the above research results, it can be found that the taking behaviour of the elderly in the kitchen space is both frequent and difficult. With the degeneration of bones and joints of elderly users, osteoporosis and other problems occur frequently, it is of great value to study the executive ability of the elderly during the holding process, that is, to explore the influence of cabinet height on the actual joint angles of different parts of the elderly’s body. The proportion of elderly women participating in kitchen activities is significantly higher than that of men, with the L-shaped layout being the most common kitchen configuration in elderly households. When performing kitchen tasks, the lower back and legs are the most frequently reported areas of discomfort among the elderly. The primary causes of lower back discomfort are washing, cutting vegetables, and cleaning, while leg discomfort is mainly due to washing, cooking, cleaning, and retrieving items from lower cabinets. For the elderly, the difficulty ranking of various kitchen tasks is as follows: retrieving items from upper cabinets > retrieving items from lower cabinets > cooking > cleaning > cutting vegetables > washing vegetables. This indicates that storage tasks are more challenging than daily tasks such as washing, cutting, cooking, and cleaning, and the difficulty of retrieving items from lower cabinets increases with age.

### Behavioural posture assessment for retrieving objects

**Fig. 1:**
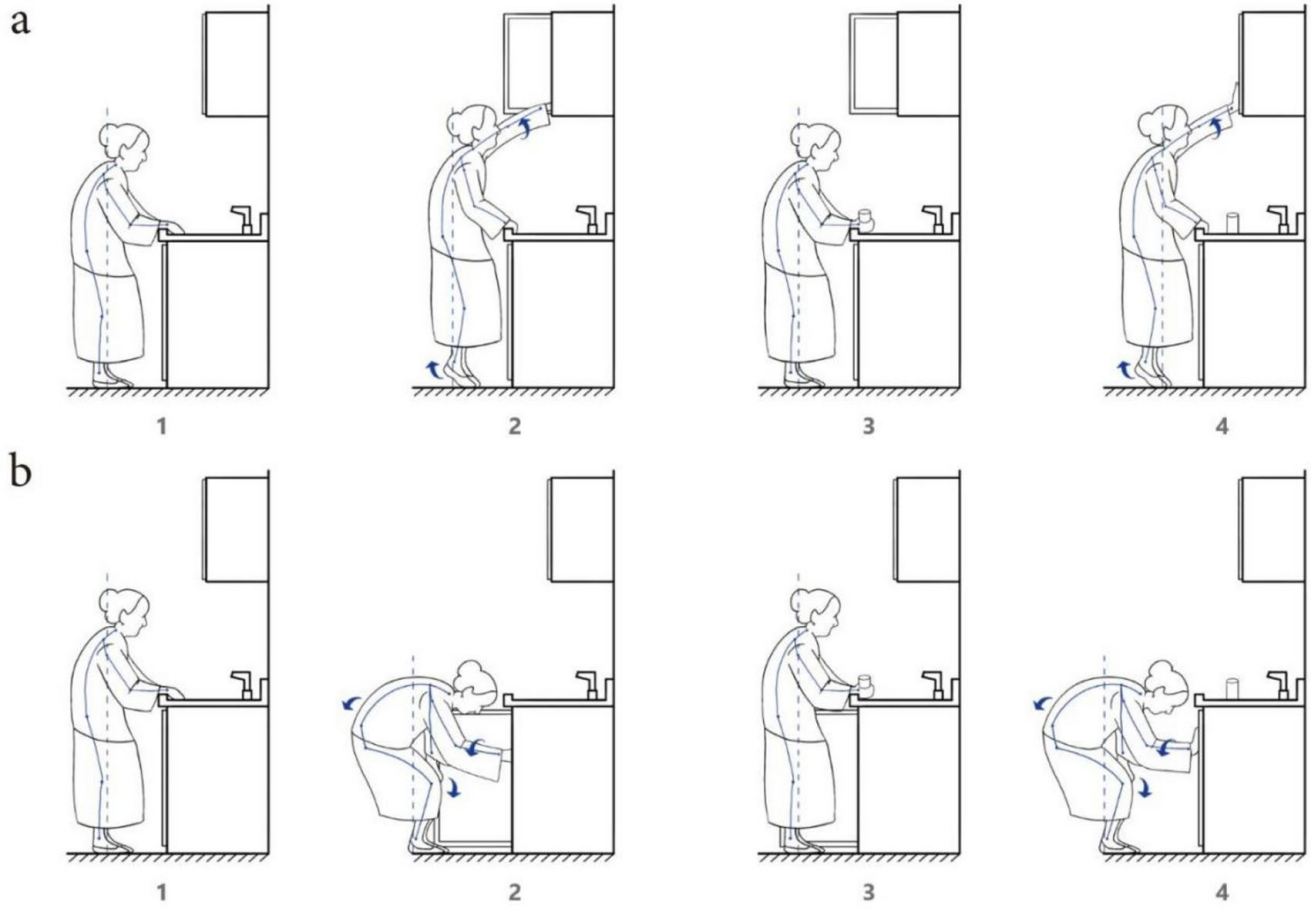
Behaviour flow of the elderly taking objects. Among them, (**a**) describes the behaviour process of the elderly taking high objects, and (**b**) describes the behaviour process of the elderly taking low objects.

According to field research and user interviews, etc., the action process of taking items from the upper cabinet and the action process of taking items from the floor cabinet is summarised as shown in Fig. 1. Analysis of the behaviour of picking up items from the cabinet: first lean forward to get close to the cabinet, then put your feet or hands on the operating table, then reach out to open the cabinet door, find the location of the item to complete the picking behaviour, and finally return to the original posture. Analysis of the behaviour of taking items from the base cabinet: First, hold the operating table with your hand, then bend down to hold the CITIC, then reach out to open the cabinet door, and find the location of the item to complete the picking behaviour, and finally return to the original posture. For the elderly group, the behaviour of bending over and stepping their feet makes it easy for them to lower their control over their centre of gravity, which is extremely prone to accidents.

### Construction of behavioural competency assessment framework

The OpenPose model is widely used in academia and industry for its high precision and reliability. It can accurately detect and identify key points of the human body, including the head, shoulders, elbows, wrists, hips, knees, and ankles. Moreover, OpenPose’s real-time processing capability allows for the quick detection of human postures in video streams, which is extremely useful for real-time monitoring and recording of participants’ movements during experiments. In recent years, many new pose estimation algorithms have been developed and have shown excellent performance and capabilities. However, OpenPose has been extensively validated in multiple studies and practical applications, and its stability and reliability have been well-proven. For this study, using a mature and widely recognized algorithm ensures the repeatability and credibility of the experimental results. Additionally, since we used a 2D webcam for data collection, OpenPose’s excellent performance in processing 2D image data makes it suitable for providing accurate posture estimation results in a single-camera setup. Furthermore, as an open-source project, OpenPose allows researchers to customize and extend it as needed, enabling the rapid setup of experimental platforms and the processing and analysis of data within limited time and resources. Its flexibility and extensibility make it an ideal tool for human posture analysis research. Certainly, we also recognize that with the advancement of technology, newer algorithms may offer higher accuracy and more powerful features. In future research, we plan to explore and compare other emerging pose estimation algorithms to further enhance the accuracy and reliability of our study. For example, improvements in pose estimation accuracy by AlphaPose and HRNet, as well as the real-time performance of BlazePose on mobile devices, are worth further exploration and application.

**Fig. 2:**
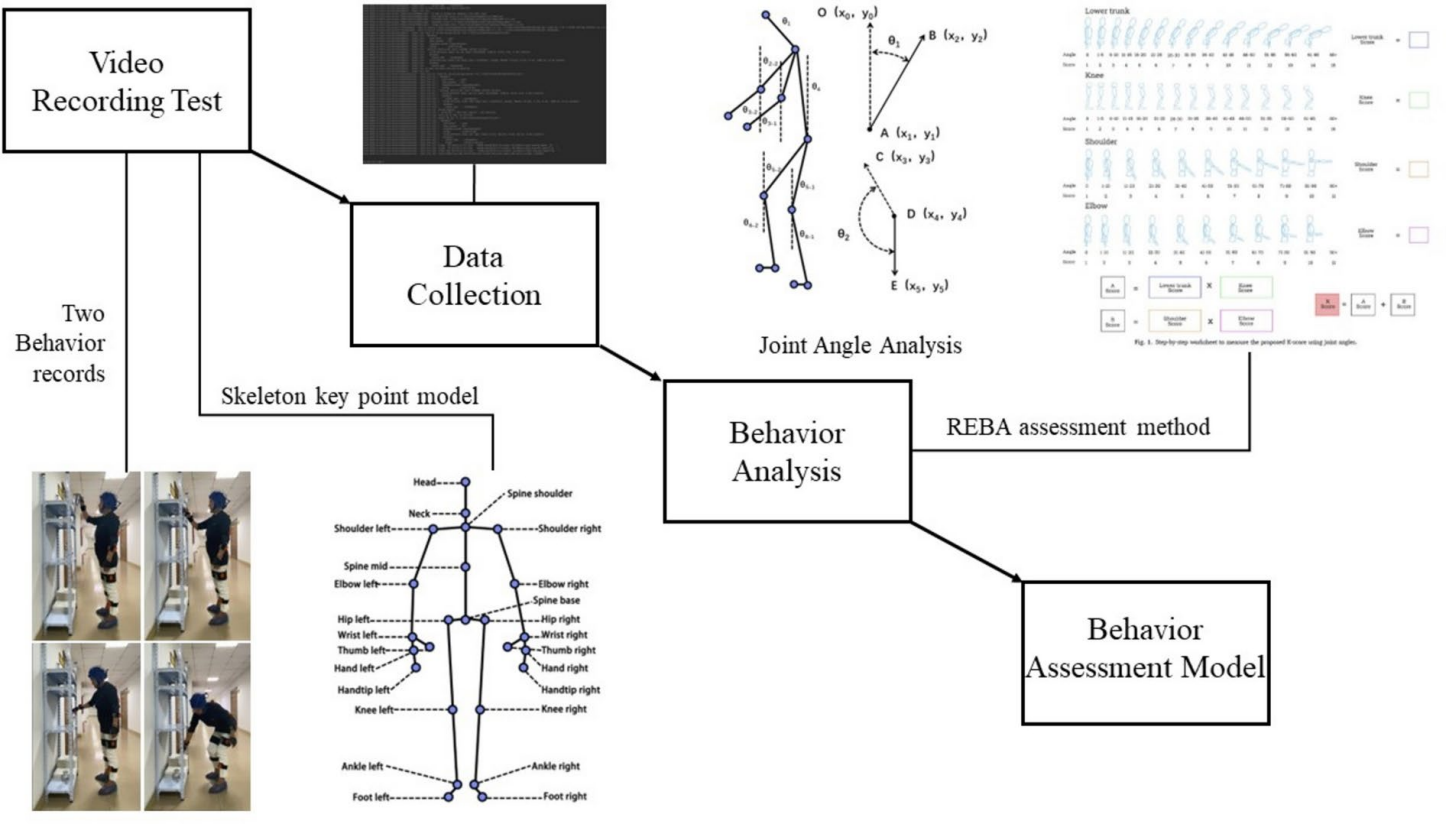
Behavioural capacity assessment model construction process.

The main network structure of OpenPose uses the VGG convolutional neural network as the backbone and employs two branch subnetworks to regress joint positions (S) and the direction of pixels in the skeleton (L). These branch networks operate iteratively across multiple stages, with each stage computing a loss function and then concatenating L, S, and the original image features extracted by the VGG convolutional neural network before proceeding to the next stage of training. Although OpenPose cannot directly obtain joint angle information, it can calculate the corresponding angles from the relative positions of key points. For instance, the neck flexion angle θ1 is the angle between the neck-head vector AB and the anti-gravity vector AO. The angle θ1 can be derived from the relative coordinates of points A, B, and O, as illustrated in the ‘Joint Angle Analysis’ schematic in Fig. 2. In our study, wrist angles were determined using the 2D positions of keypoints provided by the OpenPose model. Specifically, OpenPose gives us the coordinates of various hand and wrist keypoints in a two-dimensional plane. We calculated the vector from the wrist keypoint to the hand keypoints. Using these vectors, we determined the angles by computing the arccosine of the dot product of these vectors normalized by their magnitudes.

The REBA method is relatively simple and easy to use. By observing postures and assigning scores, it is easy to understand and apply. It provides a comprehensive assessment of whole-body posture and muscle usage, making it suitable for various work scenarios. It does not require complex equipment or specialized knowledge, and its simplicity allows for quick assessments, making it suitable for large-scale data collection and analysis. Additionally, REBA is widely used in ergonomic studies, and its assessment results have high comparability and practical application value. In our study, the primary goal was to evaluate the postures and workload of elderly individuals during kitchen activities. Given the specific characteristics of elderly users and the limitations of our experimental resources, we ultimately chose the REBA method. Firstly, elderly participants may not be able to maintain complex postures or cooperate with complicated equipment for extended periods during the experiment. Considering the special nature of the participants and the feasibility of practical operations, the REBA method proved to be more efficient in on-site implementation and data collection, effectively reducing the burden on elderly users. Secondly, the postures involved in kitchen activities for the elderly require coordination and use of multiple body parts. The REBA method offers a comprehensive evaluation of whole-body postures, not just the load on a single joint. As our study focuses not only on the load of a single joint but also on the overall working posture and muscle usage, the REBA method can more fully reflect the risks and discomfort experienced by the elderly during kitchen activities. Furthermore, our laboratory resources are limited, and the complex equipment and high costs required for biomechanical modeling exceed our experimental conditions. The REBA method, while ensuring the accuracy of the assessment, is more practical and feasible. Nonetheless, we also recognize the advantages of biomechanical models in precisely calculating joint loads. In future research, we plan to incorporate more biomechanical indicators and models to further analyze the biomechanical characteristics of elderly individuals during kitchen activities.

Using the OpenPose model and the REBA method, a WMSDs risk assessment framework for manual handling operations is constructed, as shown in Fig. 2. Apply the scoring content of REBA to the bone data processing stage, carry out risk classification on the final total score of posture, realise the output of the joint angle and output the total score of REBA of each part of the body at the same time, and generate the joint angle that changes continuously with the number of video image frames, REBA total score and the risk level curve of the operation posture. After field investigation, it was found that the neck and wrist of the lifting movement were in a relatively static state, and the three pre-input variables (load, activity frequency, and hand tools) were tentatively set to 0, and the test action was the behaviour of carrying heavy objects. The occupational posture risk assessment framework is mainly composed of four parts, namely, image and data acquisition, human skeleton recognition based on OpenPose, operation posture risk calculation based on REBA method, and generation of ergonomic risk assessment report. The evaluation process will be discussed in detail later, mainly including the calculation of human body posture angle, REBA score and determination of WMSDs risk level.

## Methods

### Experimental participants

The experimental subjects were recruited from the typical representative self-care elderly group in the Yangtze River Delta region. Members of our team contacted the relevant retirees by phone. According to specific needs, our team randomly selected 21 eligible people from the male and female groups. All subjects were right-handed, had no physical disabilities, had the ability to take care of themselves independently, and performed light daily activities. Data such as height and weight of the subjects were obtained before the test. Among the 42 participants, there were 21 males (mean standard deviation = 64.64 ± 5.61) and 21 females (mean standard deviation = 65.72 ± 4.47), all aged between 60 and 75 years (mean standard deviation = 65.18 ± 5.04). The participants in this study were self-care elderly individuals with relatively good functional abilities. Therefore, no external support, such as holding onto cabinets or using walkers, was employed during the experiment. The focus of the experimental design was to evaluate the posture and retrieval behaviors of these self-care elderly individuals without the use of assistive devices. The basic demographic data of all participants are shown in Table 4. The research protocol was approved by the Ethics Committee of the Science and Technology Department of Nanjing Forestry University (Jiangsu Province, China). Before the test, the elderly subjects were all in good health, without fatigue/sickness and mood swings. Before the experiment, all subjects read and signed the informed consent form, and a certain experimental reward was given at the end of the experiment.

**Table 4:** Basic conditions of the subjects tested.

Item	Female elderly subjects (n = 21)				Male elderly subjects (n = 21)			
	Age	Height/m	Weight/kg	BMI index	Age	Height/m	Weight/kg	BMI index
Mean	65.72	1.64	68.08	25.15	64.64	1.65	66.99	24.38
SD	4.47	0.08	9.13	2.67	5.61	0.08	9.24	2.02
Max	75.00	1.68	81.50	30.74	69.00	1.81	76.00	28.12
Min	60.00	1.50	44.00	19.55	64.00	1.66	65.00	20.87

### Measured data

**Fig. 3:**
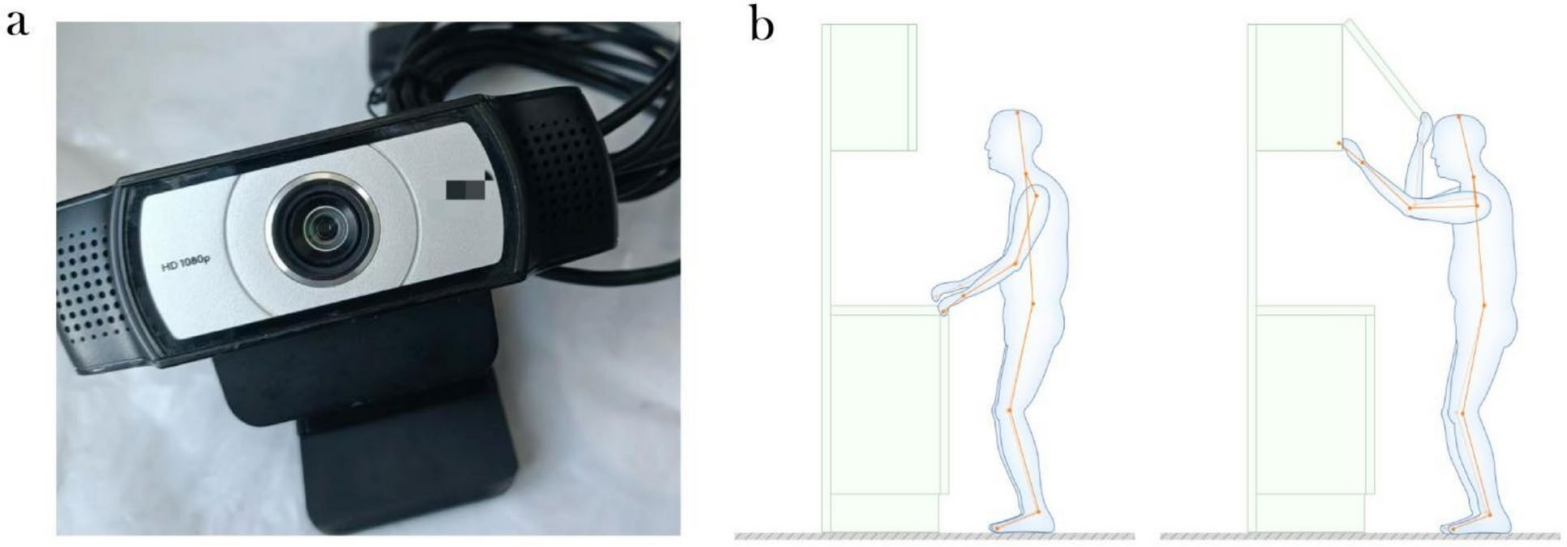
Experimental equipment.

This experiment uses the Logitech C930C video capture device, which is light and easy to carry, and the shooting angle can reach 90°, without missing every detail of the behaviour of the observed. It supports full HD 1080p image quality (30 fps), clearly displaying non-verbal information such as the subjects’ expressions and movements. The schematic of the footage captured by the equipment during the experiment is shown in Fig. 3b. At the same time, the device comes with dual omnidirectional microphones, which can clearly and accurately record the words of the observed person, as shown in Fig. 3a. The entire experimental environment is an air-conditioned, controllable indoor space with stable temperature and humidity, good lighting, and no noise. Participants were in good health and had no major diseases.

**Fig. 4:**
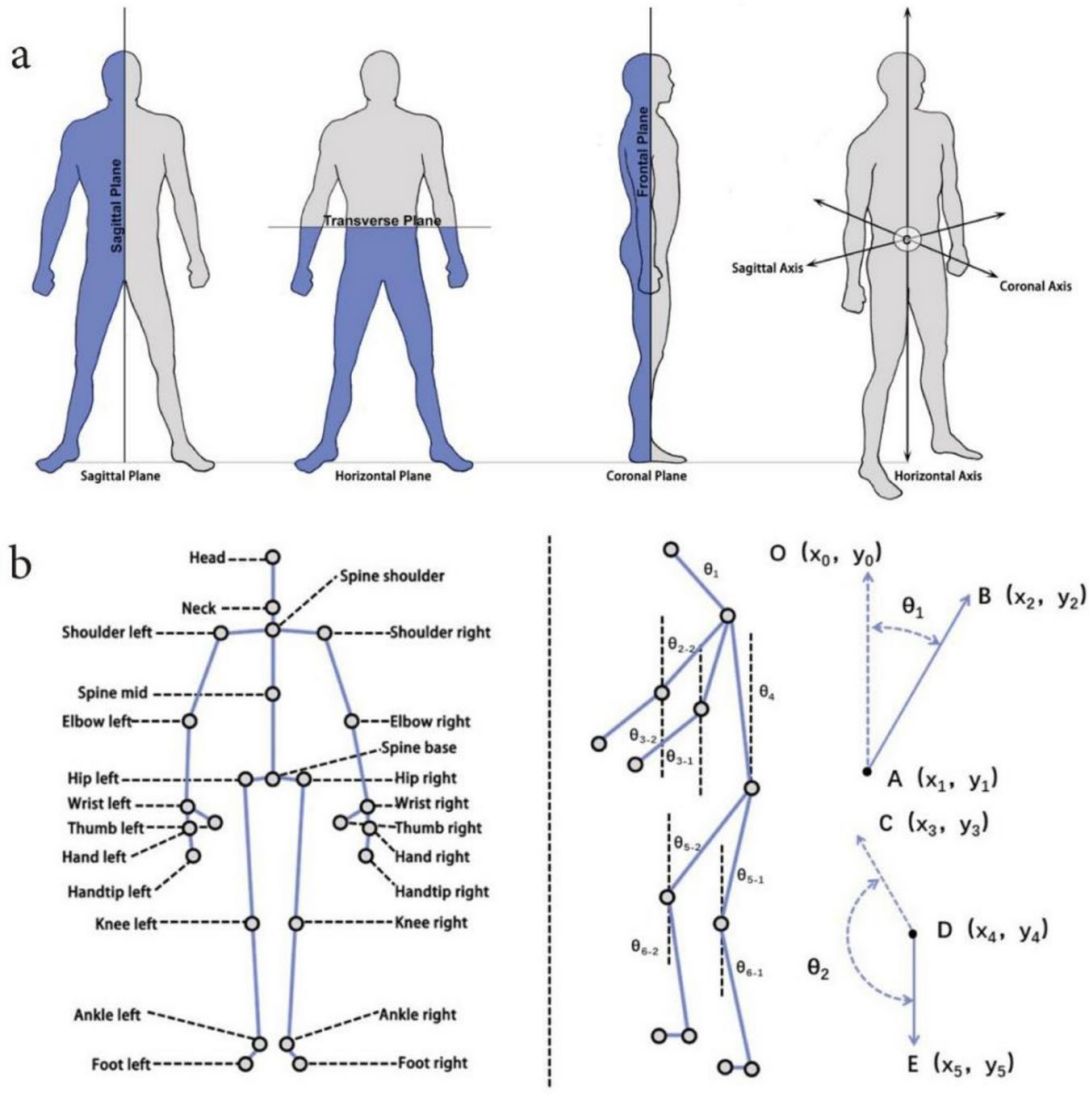
(**a**) Human motion plane details. (**b**) Details of key points of joint movement.

In sports anatomy, in order to meet the needs of observation from different directions, it is stipulated that the human body contains three motion planes: sagittal plane, horizontal plane, and coronal plane, that is, three views of the human body^54^, as shown in Fig. 4a. The sagittal plane is the front-to-back direction, the coronal plane is the left–right direction, and the horizontal plane is parallel to the ground plane. Comparing the evaluation accuracy of the key points of the behaviour of the elderly in the sagittal plane, horizontal plane, and coronal plane, it is concluded that the key data of the human body obtained by measuring the sagittal plane is more accurate in evaluating the user’s executive ability. The change of each joint of the body on the sagittal plane when the fetching behaviour is completed, in order to further explore the optimal range of fetching objects on the sagittal plane for the elderly. In the data analysis, this article only considers the changes in the flexion/extension angles of each joint of the human body, and does not involve the angle changes in adduction/abduction (Abduction/Adduction) and internal/external rotation (Internal/External Rotation), joint activities The schematic diagram is shown in Fig. 4b.

### Rapid entire body assessment

We use the REBA method to evaluate. The use behaviour stage is subdivided into nine use behaviour processes, and the posture load in the use behaviour process is evaluated from the joint angles of nine static postures. The scoring characteristics of REBA method are that the lower limbs of the body are dominant and the upper limbs are assisted to get the score A and B, and the score C is obtained by looking up the table of the score matrix, with the activity score as the additional score^55^. Secondly, the muscle usage, load and exertion are different in each behaviour stage, so it is necessary to add the muscle usage score, load or exertion score on the basis of score A and score B when calculating the total score^56^. In the REBA evaluation method, the lower limbs include neck, trunk and legs. The posture of the neck is divided into forward and backward, with the inclination angle of the neck as the scoring variable, and the trunk is the same, and the legs are scored according to the state, while considering the bending angle of the knees^57^. Upper limbs include upper arm, forearm and wrist. The angle quantification of upper arm is the included angle between arm and trunk, forearm is the elbow bending angle, that is, the included angle between forearm and upper arm, and wrist is the included angle between hand and forearm^58^. The evaluation standard of the upper arm posture gradually increases from 1 to 4. The greater the lifting angle, the more points are scored. When the angle between the upper arm and the trunk is greater than 90 or between 90 and 180, the unified basic score is 4. The situations that increase the score include shoulder lifting, arm twisting and abduction, but when the arm has external support, it needs to be reduced by 1 point. The elbow bending angles are divided into 60–100°, 0–60° and above 100° , and the scores are 1 point and 2 points respectively, and the score is increased by 1 point when the front arm moves in or out. When the wrist tilt angle is between 0 and 15, increase 1 point, and increase 2 points beyond this range. In the process of getting up, the neck of the elderly is leaning forward, with 20 as the dividing line, and 2 points will be added if it exceeds 20. The bending angle of the trunk is also designed within the range of 1–4 points, and when the human body is in an upright position, no points are given. Finally, the leg posture, the state of the leg is divided into two types, one is to increase 1 point when carrying weight on both sides, walking or sitting, and the other is to increase 2 points when carrying weight on one side, carrying weight periodically or in an unstable posture. At the same time, considering the knee bending angle, when the angle is between 120 and 150, it will be increased by 1 point, and when the bending amplitude is gradually increased and the leg included angle is within 120, it will be increased by 2 points^59,60^.

**Fig. 5:**
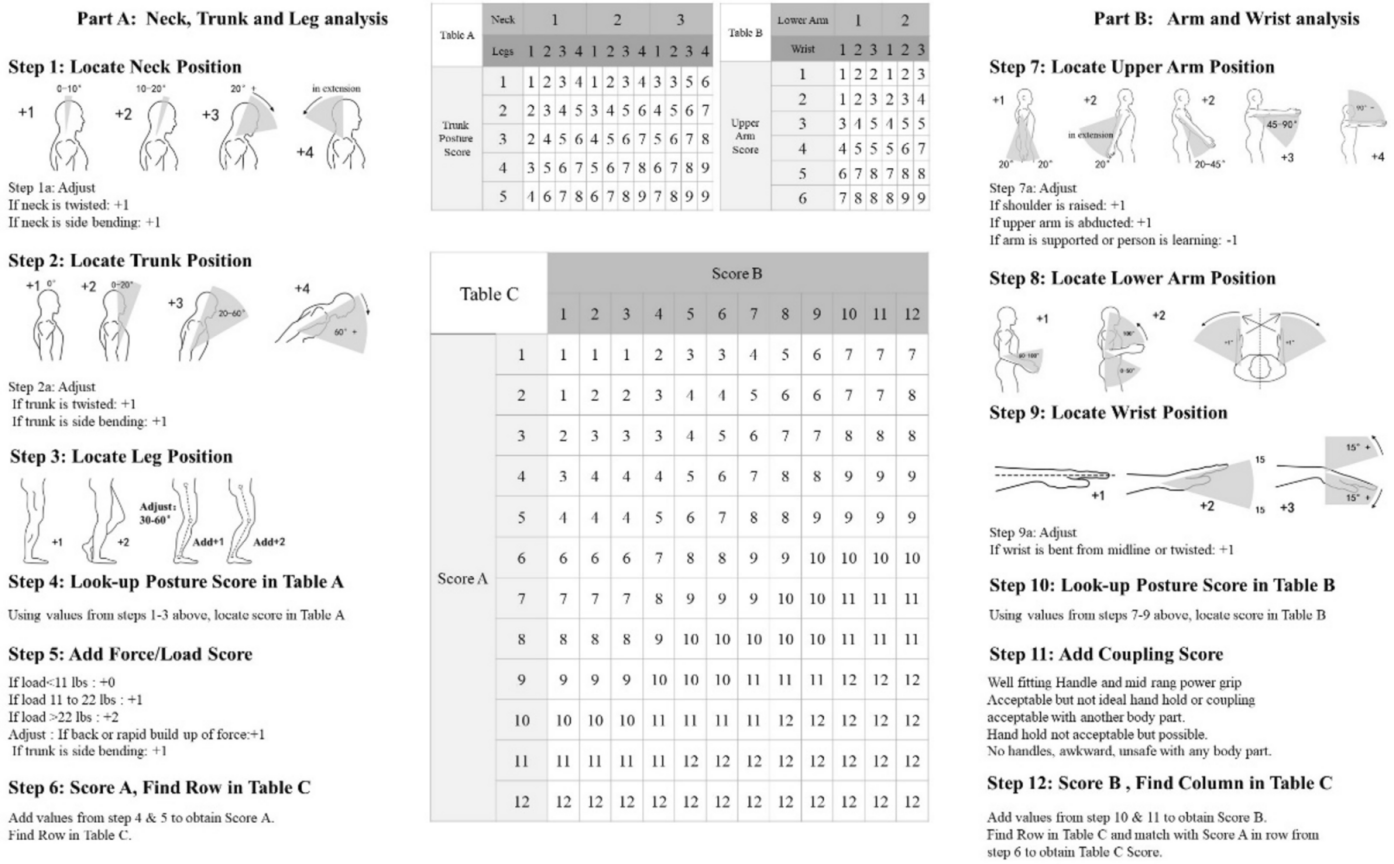
REBA assessment worksheet.

The main process of REBA method is shown in Fig. 5. The details are as follows: The body parts are divided into two groups. Group A includes trunk, neck and legs, while Group B includes upper arms, forearms and wrists. They are scored according to the REBA load level. Each body part is based on one score according to different postures, and gradually increases according to the scoring standard, and the scoring range is between 1 and 4. At the same time, set additional scores, determine muscle use and strength scores based on field investigation and observation results, get score A and score B, and then get the total score, namely score C, by looking up the table. On this basis, it is necessary to consider the activity score. The specific content of the activity type includes that one or more body parts are still for more than 1 min; Repeated small-scale actions, such as repeating more than 4 times per minute (excluding walking); There are three categories: posture changes rapidly and widely or support instability caused by actions, with an additional score of 1 point for each category. Add the activity score and the score c to get the total score of REBA, ranging from 1 to 15 points, and then determine the risk level and action level.

Based on the above analysis, the whole-body evaluation method for getting up behaviour is designed with scores, and the corresponding risk levels and action levels are divided, as shown in Table 5. The highest REBA score is 15 points, and the corresponding scores are divided according to the five action levels to evaluate the risk level of the posture. The risk level is divided into negligible, low, medium, high and very high, and above medium risk requires action to improve. When the REBA score is 1, the risk level is negligible, which means that the risk is very small and almost negligible; when the REBA score is between 2 and 3 points, the risk level is low and within the acceptable range; when the REBA score is When the value is between 4 and 7, the risk level is medium, and improvement is required at this time; when the REBA score is between 8 and 10, the risk level is high, and immediate action is required; when the REBA score is between 11 and 15 Between , the risk level is very high, and this risk needs to be taken seriously and acted upon^61,62^.

**Table 5:** REBA-based approach risk level and posture assessment.

Action level	REBA score	Risk level	Posture assessment and action
0	1	Ignore not count	The risk is very small, almost negligible
1	2–3	Low	The risk is very low and within acceptable limits
2	4–7	Middle	Risk is moderate and needs improvement
3	8–10	High	Very high risk, act now
4	11–15	Very high	Very high risk, act now

### Experiment procedure

**Fig. 6:**
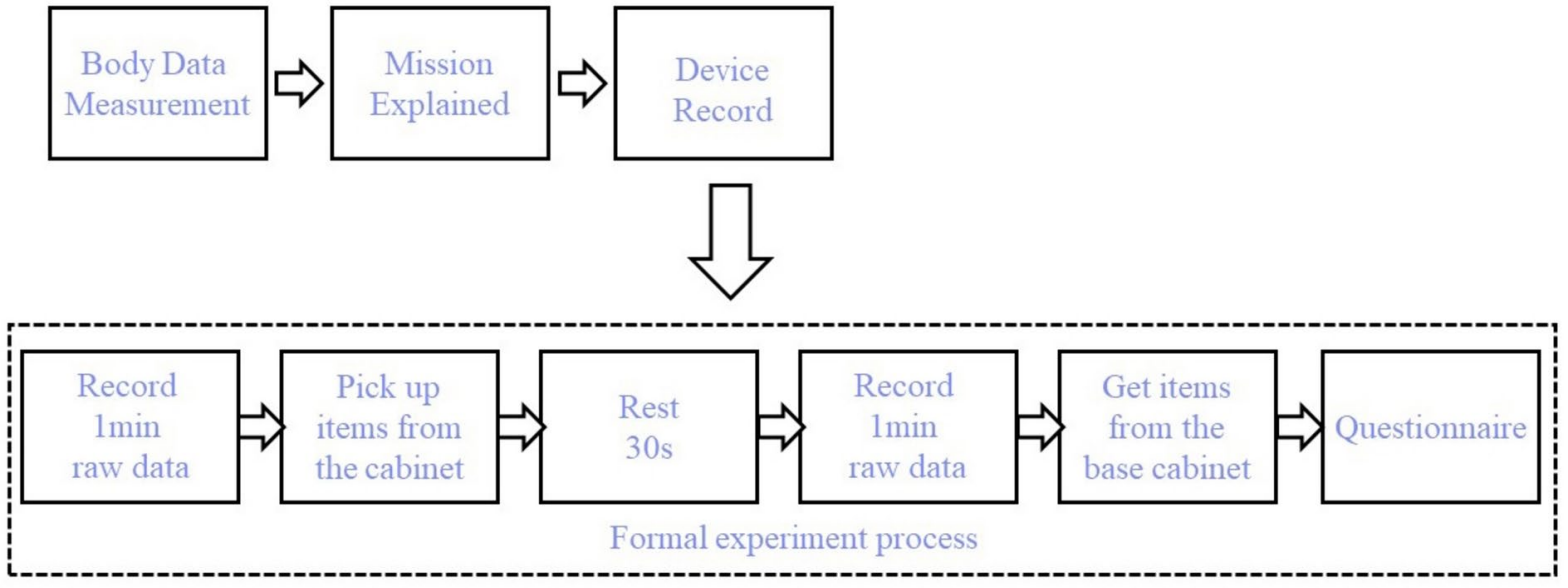
Experiment procedure.

The purpose of this experiment is to collect and record the action postures and behavioural states of the elderly when they take objects from the base cabinet and upper cabinet objects of different heights. In this study, kitchen utensils in a straight layout and self-made chests of drawers of different levels were arranged as the experimental scene, as shown in the Fig. 6. The specific experimental process is described as follows: First, before the experiment, the main experimenter must collect basic information such as the age, height and weight of the subjects, and introduce the purpose, significance and main content of the experiment to the subjects. Then the subject stood in the designated area. The main tester needs to adjust the Logitech C930C video capture device in advance and use a tripod to fix it at a position where the overall situation can be captured without affecting the behaviour of the observed person. The angle of view of the camera is adjusted to 90° Large wide angle ensures a wider collection angle for later observation. Afterwards, the experimental tasks were formally started: (1) Take the object at the height of the cabinet: firstly, let the subject stand still and upright on the spot for 10 s, then ask the subject to bend down to pick up a cylinder with a weight of 100 g on the marked point on the ground, After the completion of the experiment, the subjects remained in the standing still and upright state for 10 s according to the command of the experimenter. The subjects rested for 2 min, and communicated with the experimenter about the behaviour of taking the items from the base cabinet before proceeding to the follow-up task. (2) Take objects of different heights from the upper cabinet: firstly, let the subjects stand still and upright on the spot for 10 s, and then ask the subjects to take the cylinders with a weight of 100 g from the upper cabinet in order of heights of 120 cm, 130 cm, The laminates of 140 cm and 150 cm were taken out and placed on the kitchen countertop with a height of 85 cm. After taking them all out, the subjects stood still and upright for 10 s. Choose the standard source of height After the experiment, the subjects were asked about their behavioural feelings when taking objects of different heights.

### Ethics approval and consent to participate

For experiments involving human participants, informed consent has been obtained from all subjects (all adults) in this study. It is important to note that the participation of all participants in the study is completely voluntary. They have the option to withdraw from the study at any time. Furthermore, the data collected will be used solely for academic purposes. In accordance with the Document No. 1 of Science and Technology Department of Nanjing Forestry University [2021], our research obtained ethical approval from the Ethics Committee of Nanjing Forestry University. All methods were carried out in accordance with relevant guidelines and regulations.

## Results

### Behavioural observation analysis results

#### Behaviour analysis of taking items from the cabinet

**Fig. 7:**
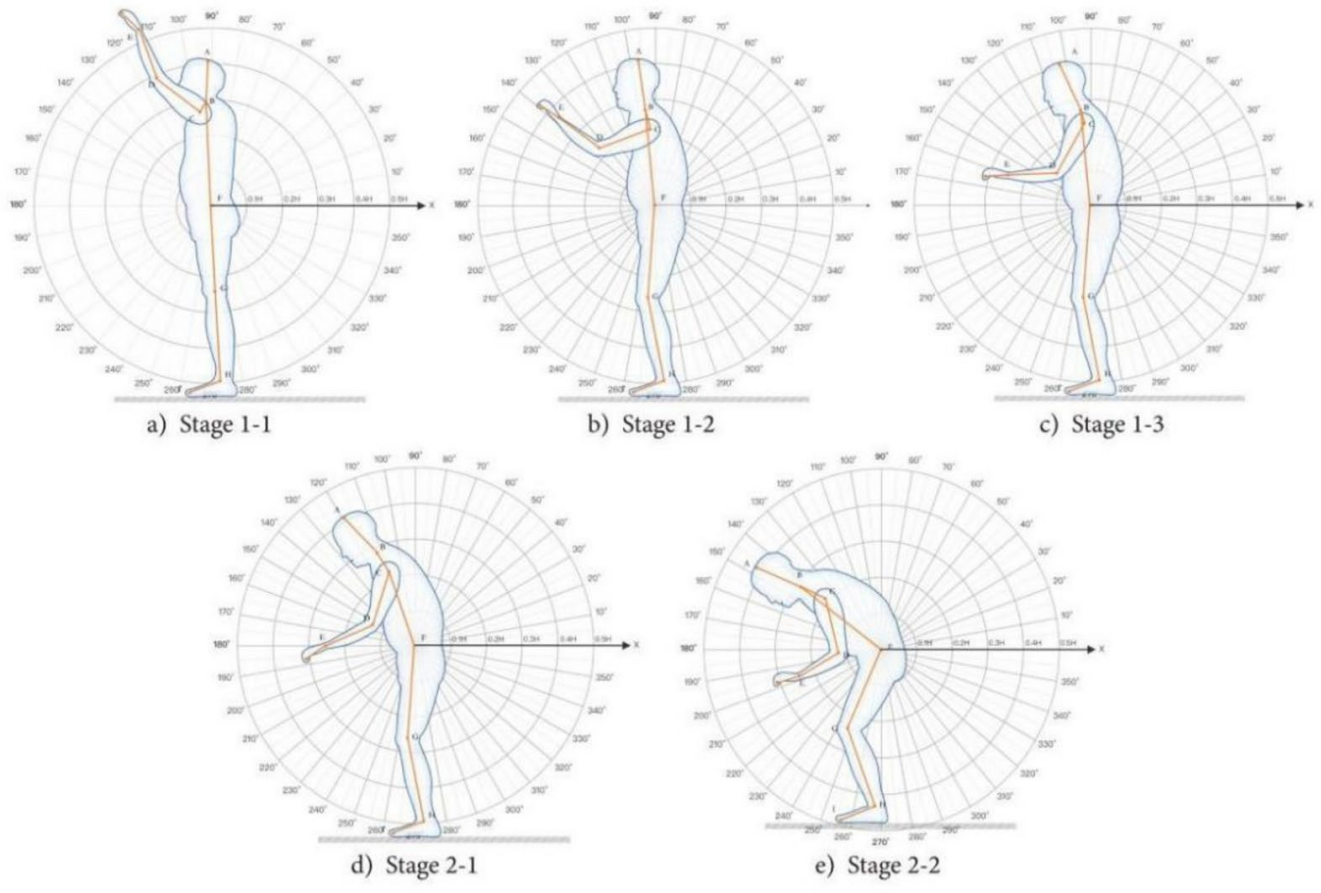
Take-use behaviour stage. The neck offset angle θ1, the upper arm offset angle θ2, the lower arm offset angle θ3, the trunk offset angle θ4, Thigh offset angle θ5, thigh and calf included angle θ6, and calf ground included angle θ7.

In the daily life activities of older persons, there are often high-frequency taking actions. With the deterioration of skeletal joints of elderly users, osteoporosis and other problems frequently occur, so it is of great significance to study the actual joint and muscle contraction tension in the process of taking^63,64^. Actually, simulate the scene of the elderly users taking things, and set five heights to ensure that they can take the same heavy object, as shown in Fig. 7, so as to test the taking ability and grasping ability of the elderly subjects.

When picking up items at the same height from the upper cabinet, the peak flexion angle of the right shoulder of women is greater than that of men, and this difference is caused by the difference in height between men and women. The taller the subject, the smaller the peak flexion angle of the shoulder on the operating side when picking up the items from the cabinet. When the female subjects picked up the items on the cabinet, the flexion angle of the right shoulder would increase with the increase of the height of the items. When the male subjects picked up objects on the table with a height of 120 cm and 130 cm, the peak value of the flexion angle of the right shoulder was not much different; Compared with the peak value of flexion angle at 120 cm and 130 cm, the peak value increased significantly.

The ‘retrieving behavior’ study primarily focuses on the process of retrieving items from high places. Data were recorded using a Logitech C930C camera, and the OpenPose algorithm was used to measure the relative positions of key points and calculate the corresponding angle values. Analysis of the actual measurements with Origin software yielded the following data:

- Neck: range of motion θ = {θ|2.489° ≤ θ < 11.888°}.
- The upper arm of the trunk: range of motion θ1 = {θ|89.879° ≤ θ < 89.948°}; range of motion θ2 = {θ|89.905° ≤ θ < 89.951°}.
- Elbow bend: range of motion θ1 = {θ|32.717° ≤ θ < 172.446°}; range of motion θ2 = {θ|38.445° ≤ θ < 171.422°}.
- Trunk flexion: range of motion θ = {θ|145.539° ≤ θ < 177.247°}.
- Trunk and thigh: range of motion θ1 = {θ|3.190° ≤ θ < 105.319°}; range of motion θ2 = {θ|23.326° ≤ θ < 164.603°}.
- Thigh and calf: range of motion θ1 = {θ|2.752° ≤ θ < 139.085°}; range of motion θ2 = {θ|0.151° ≤ θ < 7.548°}.
- Ground part of calf: range of motion θ1 = {θ|1.245° ≤ θ < 174.236°}; range of motion θ2 = {θ|46.802° ≤ θ < 65.912°}. Details are shown in Table 6.

**Table 6:** The range of inclination angles of each body part when taking the high objects.

Homework tasks	Body part angle	θ1	θ2
Take high objects	Neck	2.489° ≤ θ < 11.888°	
	The upper arm of the trunk	89.879° ≤ θ < 89.948°	89.905° ≤ θ < 89.951°
	Elbow bend	32.717° ≤ θ < 172.446°	38.445° ≤ θ < 171.422°
	Trunk flexion	145.539° ≤ θ < 177.247°	
	Trunk and thigh	3.190° ≤ θ < 105.319°	23.326° ≤ θ < 164.603°
	Thigh and calf	2.752° ≤ θ < 139.085°	0.151° ≤ θ < 7.548°
	Ground part of calf	1.245° ≤ θ < 174.236°	46.802° ≤ θ < 65.912°

#### Behaviour analysis of taking items from the base cabinet

**Fig. 8:**
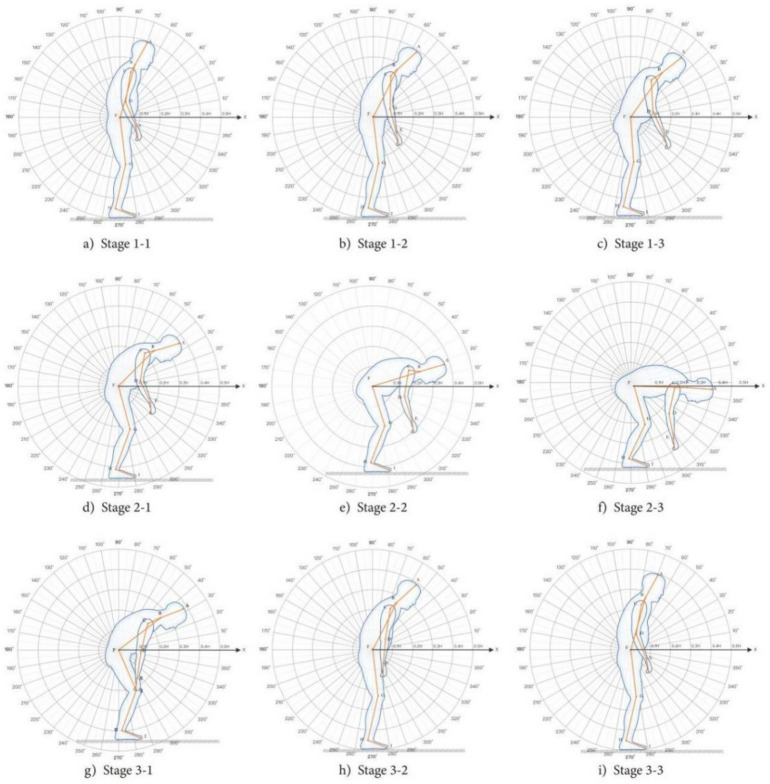
Bent over behaviour stage. The neck offset angle θ1, the upper arm offset angle θ2, the lower arm offset angle θ3, the trunk offset angle θ4, Thigh offset angle θ5, thigh and calf included angle θ6, and calf ground included angle θ7.

With the aging of elderly users, physiological problems become increasingly prominent. The time and frequency of elderly users’ bending have obviously increased. With the increasing frequency of bending, there is bound to be an increase in the security risks of getting up, and the risk is greatly improved^65,66^. According to the summary of experimental samples, the behaviour process of bending over is shown in Fig. 8.

When bending over to pick up items from the base cabinet, the peak flexion angles of the hip and knee joints on the operating hand were greater than those on the non-operating hand. When picking up items from the base cabinet, the maximum flexion angles of the left and right hip joints and knee joints will decrease as the height of the items increases. angle. When picking up objects from the floor cabinet, the peak flexion angles of the left and right hips of women were significantly greater than those of men. This difference is caused by the difference in the habits of men and women, not the difference in height.

The ‘bending behavior’ study primarily focuses on the process of bending and standing up. Data were recorded using a Logitech C930C camera, and the OpenPose algorithm was used to measure the relative positions of key points and calculate the corresponding angle values. Analysis of the actual measurements with Origin software yielded the following data:

- Neck: range of motion θ = {θ|8.130° ≤ θ < 113.962°}.
- The upper arm of the trunk: range of motion θ1 = {θ|89.835° ≤ θ < 89.891°}; range of motion θ2 = {θ|89.849° ≤ θ < 89.914°}.
- Elbow bend: range of motion θ1 = {θ|54.906° ≤ θ < 178.354°}; range of motion θ2 = {θ|46.277° ≤ θ < 139.398°}.
- Trunk flexion: range of motion θ = {θ|131.583° ≤ θ < 143°}.
- Trunk and thigh: range of motion θ1 = {θ|2.750° ≤ θ < 127.873°}; range of motion θ2 = {θ|0.367° ≤ θ < 153.488°}.
- Thigh and calf: range of motion θ1 = {θ|2.403° ≤ θ < 179.876°}; range of motion θ2 = {θ|24.074° ≤ θ < 180°}.
- Ground part of calf: range of motion θ1 = {θ|8.130° ≤ θ < 120.963°}; range of motion θ2 = {θ|77.275° ≤ θ < 171.347°}. Details are shown in Table 7.

**Table 7:** The range of inclination angles of each body part when taking the low objects.

Homework tasks	Body part angle	θ1	θ2
Take low objects	Neck	8.130° ≤ θ < 113.962°	
	The upper arm of the trunk	89.835° ≤ θ < 89.891°	89.849° ≤ θ < 89.914°
	Elbow bend	54.906° ≤ θ < 178.354°	46.277° ≤ θ < 139.398°
	Trunk flexion	131.583° ≤ θ < 143°	
	Trunk and thigh	2.750° ≤ θ < 127.873°	0.367° ≤ θ < 153.488°
	Thigh and calf	24.03° ≤ θ < 179.876°	24.074° ≤ θ < 180°
	Ground part of calf	8.130° ≤ θ < 120.963°	77.275° ≤ θ < 171.347°

### REBA scoring results

As introduced above, we used the REBA method for evaluation. The usage behavior stage has been refined into several specific postures. These postures were recorded by a Logitech C930C camera. The video data were analyzed, and continuous movements were broken down into a series of individual static postures, each corresponding to a specific set of key point locations. As shown in Figs. 7 and 8, OpenPose was chosen as the posture recognition tool. It automatically identifies and marks skeletal key points of the body, including the head, neck, shoulders, elbows, wrists, hips, knees, and ankles. The postural load during the usage behavior process was evaluated using the joint angles of several static postures.

Based on the subdivision scores of different body parts and joint angle changes, as well as the scores of external load and muscle exertion during the movement, the score A and score B are obtained by querying the score matrix table^67,68^. The score A mainly evaluates the posture of human lower limbs, including the trunk, neck and legs. The trunk determines the number of rows, and the neck and legs jointly determine the number of columns. For example, when the trunk is 3 points, the neck is 1 point, and the legs are 2 points, the score A is 4. At the same time, if the force is exerted by an external load, the score will be added according to the size of the force^69^. The score B mainly evaluates the posture of human upper limbs, including the upper arm, elbow and wrist. The upper arm determines the number of rows and the elbow and wrist determine the number of columns. For example, when the upper arm is 4, the elbow is 2 and the wrist is 3, the score B is 7. Finally, combining score A and score B, the total score of REBA is obtained through the matrix scale of score C.

Analyze the behaviour process of users taking items in the cupboard. When taking the articles from the bottom cabinet, the behaviour stage 1 is the preparation stage of the elderly subjects, the behaviour stage 2 is the bending stage of the elderly subjects, and the behaviour stage 3 is the standing stage of the elderly subjects. When picking up the articles on the counter, the behaviour stage 1 is the stage of raising the hand of the elderly subjects, and the behaviour stage 2 is the stage of bending over the elderly subjects. Each behaviour stage is subdivided into three small behaviour postures, and the corresponding risk level is obtained through the evaluation of REBA method. According to the REBA method, a series of factors, including body posture, load, muscle use, etc., are detected, and the corresponding scores of each factor are obtained through the score query table, and the total score of REBA is accumulated. The greater the score, the higher the risk of this posture. The evaluation process of REBA method for taking the behaviour and posture load of objects in the cupboard is as follows: firstly, the posture of two groups of body parts is scored according to the scoring standard, and the load score and muscle use score are added at the same time; Secondly, look up the table according to the scores of each part to get a score A and a score B; Finally, combined with the activity score, the total score of REBA is obtained, and the corresponding action level and risk level are given through the risk level table, so as to evaluate the posture risk degree and give the measures that need to be taken to improve posture.

The scores and grades of posture load REBA evaluation method in bending behaviour stage are shown in Table 8. The scores in the table represent the average values for all participants. In the behaviour stage 1 of taking off the cabinet items, the risk level of stages 1–1 and 1–2, that is, actions A and B, is 0, and the risk level is negligible. At this time, the body posture of the elderly subjects changes slightly, so the posture load risk can be ignored. The risk levels of Action C and Action D are low risk and medium risk, respectively. In these behavioural stages, the limb changes slightly, but the whole body muscles begin to exert strength. For medium risk, we can take improvement actions and pay attention to the limb load. Action e, action f and action g are high risks, the range of trunk changes increases, and the bending angle of waist and legs increases. If these behavioural stages are repeated many times for a long time, it will increase the human load and cause unnecessary damage. Therefore, in the behaviour stages 2–2, 2–3 and 3–1, that is, bending over and getting up, we can increase the improved design or external support to help the elderly users. From the point of view of the scores of each part, the scores of trunk, legs and neck increase, and focus on the external assistance of these body parts. In the behaviour stage 3, the risk level of action H is medium risk, and the risk of action I can be ignored. In this stage, the elderly users gradually return to the upright state, from getting nervous to relaxing, and the posture load is reduced.

**Table 8:** The score and rating of the REBA for the bent over behaviour stage.

Action	Trunk	Neck	Leg	Load	Muscle use	A	Upper arm	Fore-arm	Wrist	Load	Muscle use	B	Total score	Action level	Risk level
a	1	1	1	0	0	1	1	1	1	0	0	1	1	0	Omit
b	1	2	1	0	0	1	1	1	1	0	0	1	1	0	Omit
c	2	2	1	0	1	4	1	2	1	0	0	1	3	1	Low
d	3	2	2	0	1	6	1	2	1	0	0	1	6	2	Medium
e	4	3	3	0	1	9	2	2	1	0	0	2	9	3	High
f	4	3	3	1	1	10	2	1	2	1	1	4	11	4	High
g	4	3	2	1	1	9	2	1	2	1	1	4	10	3	High
h	2	2	1	1	1	5	1	1	1	1	0	2	4	2	Medium
i	1	1	1	0	0	1	1	1	1	0	0	1	1	0	Omit

The scores and grades of the posture load REBA evaluation method for taking over-the-counter items are shown in Table 9. The scores in the table represent the average values for all participants. In the first stage of taking over-the-counter goods, the risk level of the whole stage is medium risk, and the trunk changes slightly in each stage, but the upper limb muscles begin to exert force and produce limb load, so some improvement behaviours can be taken appropriately. In the behaviour stage 2, the action D is low risk, at this time, the body posture changes slightly, and the limb load is almost negligible, while the action E is in the middle risk because of the bending of the trunk, so it can be improved appropriately.

**Table 9:** The score and rating of the REBA for the stage of taking top cabinet items.

Action	Trunk	Neck	Leg	Load	Muscle use	A	Upper arm	Fore-arm	Wrist	Load	Muscle use	B	Total score	Action level	Risk level
a	1	1	1	1	1	3	5	1	3	1	1	9	7	2	Medium
b	1	1	1	1	1	3	4	1	2	1	1	7	6	2	Medium
c	1	1	1	1	0	2	3	2	2	1	1	7	5	2	Medium
d	1	1	1	1	0	2	2	2	2	1	0	4	3	1	Low
e	2	2	2	1	1	6	2	2	2	1	0	4	7	2	Medium

## Discussion

From the above experimental results, the high-risk behaviour stages that the elderly subjects need to pay attention to in the process of taking the goods from the bottom cabinet are mainly in the behaviour stages 2–2, 2–3 and 3–1. The differences between these three stages and the adjacent stages are reflected in the changes of posture angles of trunk, legs, neck and wrist. In this state, the torso is bent, the knees are bent, the legs are stressed, the neck is forced, and the wrist is bent. Therefore, it is necessary to focus on the second half of the bending process and the first half of the rising process. The posture angle of the upper limbs changes with the change of the trunk of the main body, but there is basically no muscle use except the load of goods. From the trunk, legs and neck, the lumbar spine and knees are used in the whole process from upright to bending to upright, and the muscle load of trunk is the most obvious. With the change of angle, the physical load increases, and the risk level is also improved. In the functional design of cabinets, we should try our best to reduce the degree of limb bending of old users, or give assistance from external forces in the direction of anti-gravity to share the load of trunk angle change.

*Execution capability gradient construction*. With the decline of physical function and the change of body data threshold, elderly users will have a lot of operational ability decline or lack of ability during their behaviour^70,71^. Therefore, the angle data generated in the user’s operation behaviour experiment is used as a parameter to construct the elderly user’s operation ability model. In human body data measurement, we mainly consider standing posture and sitting posture, and measure L1 height, L2 upper arm length, L3 forearm length, L4 greater trochanter point height, L5 lower leg length, L6 eye height, L7 shoulder height, L8 elbow height, L9 hand function height, L10 arm function lifting height, L11 tibia point height, L12 arm function spreading width, L13 elbow spreading width, L14 maximum body breadth, Z1 sitting height, Z2 sitting shoulder height. Z5 sitting knee height, Z6 sitting eye height, Z7 leg plus foot height, Z8 forearm elongation before hand function, Z9 sitting lower limb length, Z10 sitting occipital protuberance height, Z11 sitting elbow height, Z12 upper limb elongation before function, Z13 elbow width, Z14 sitting hip breadth. The main 21 body key points in body angle measurement are: left hip, right hip, middle hip, top of head, right corner of mouth, neck, left shoulder, right shoulder, middle shoulder, left knee, left ankle, left corner of mouth, right elbow, right ear, nose, left wrist, left ear, left elbow, right ankle, right knee and right wrist^72,73^. Twelve angles are calculated by the above formula: neck bending angle θ1, trunk arm included angle θ2–1, trunk arm included angle θ2–2, elbow bending angle θ3–2, trunk bending angle θ4, trunk thigh angle θ5–1, trunk thigh angle θ5–2, thigh and calf included angle θ6–1, thigh and calf included angle θ6–2, leg ground included angle θ7–1, leg ground included angle θ7–2^74^. Combining them, the user behaviour model is constructed, and the mapping from user body data to key points of user bones to furniture design parameters is formed. The processing of human body data follows the sequence from two-dimensional space to three-dimensional space, and extends the traditional ergonomic measurement to the concept of human behaviour domain^75,76^.

Combined with OpenPose model and REBA evaluation data, we construct a gradient of the behaviour ability of taking cabinet items. The data recorded in the 42 videos were analyzed as a whole, and the comfort gradient data of the three parts of the subject’s neck, trunk, and knees were obtained. Origin data analysis software was used to conduct descriptive analysis of the data. The obtained mean and the standard deviation results are shown in Table 10. This data is used to support the leaning and reaching comfort gradient model for elderly users.

**Table 10:** Neck, trunk and knee comfort results.

	Degree	Normal	Easy to moderate	Moderate to laborious	The most laborious point
Neck	Mean	17°	49°	57°	60°
	SD	2.41	3.00	4.83	4.79
Trunk	Mean	1°	12°	28°	40°
	SD	0.64	1.42	2.44	3.31
Knees	Mean	179°	151°	139°	126°
	SD	0.69	1.73	2.45	3.56

Analysis of variance (ANOVA) showed that the effects of neck, as shown in Table 11, trunk and knee were all highly significant, *P* < 0.001. After the Tukey-HSD test of various factors, it was found that there were significant differences (*P* < 0.001) in the comfort values of the neck, trunk, and knees of the elderly subjects. The testers can distinguish between “easy, moderate and strenuous”, and the data are valid.

**Table 11:** Inter agent effect test.

Dependent variable	Type III sum of squares	Degrees of freedom	Mean square	F	*p*
Neck	22,972.337	3	7657.446	501.356	*p* < 0.001
Trunk	17,443.739	3	5814.580	1202.469	*p* < 0.001
Knees	30,375.623	3	10,125.208	1832.278	*p* < 0.001

**Fig. 9:**
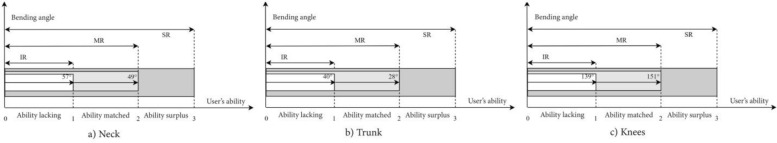
Executive ability gradient.

From this, the gradient of leaning over is obtained: (1) neck: easy range x = {x|17° ≤ x < 49°}; moderate range x = {x|49° ≤ x < 57°}; strenuous range x = {x|57° ≤ x < 60°}. (2) Trunk: easy range x = {x|1° ≤ x < 12°}; moderate range x = {x|12° ≤ x < 28°}; strenuous range x = {x|28° ≤ x < 40°}. (3) Knee: easy range x = {x|151° ≤ x < 179°}; moderate range x = {x|139° ≤ x < 151°}; strenuous range x = {x|126° ≤ x < 139}. The visualization results are shown in Fig. 9.

In this study, we chose to use computer vision technology, specifically combining the OpenPose model with the REBA method for evaluation and measurement. This choice is justified by the significant advantages of computer vision technology in several aspects. Firstly, computer vision technology has lower operational costs compared to traditional motion capture systems, as it requires only standard cameras and open-source software. This not only reduces experimental application costs but also makes the technology more accessible and widely applicable. Secondly, the automated data processing capability of computer vision technology allows for the automatic extraction and analysis of data from videos, significantly reducing the time and effort required for manual observation and data entry, thereby enhancing the efficiency and accuracy of the evaluation process. Additionally, unlike motion capture systems that require participants to wear specialized suits, computer vision technology can unobtrusively capture natural behaviors, ensuring that participants can operate in a more natural and comfortable state, resulting in more accurate and representative data. This non-invasive and easily implementable feature makes computer vision technology more flexible and widely applicable in various environments. Furthermore, computer vision technology can be easily integrated with other technologies and data sources, such as environmental sensors or wearable devices, providing comprehensive ergonomic and human factor analyses. The significant advancements in computer vision algorithms and hardware in recent years have greatly enhanced the accuracy and capabilities of these systems, making them a viable alternative to traditional methods. In summary, while motion capture systems and manual observation each have their advantages, our study uses computer vision technology due to its comprehensive benefits in cost-effectiveness, automation, non-invasiveness, versatility, and technological advancements. By leveraging these advantages, we aim to conduct comprehensive and efficient ergonomic assessments, providing valuable insights into the behaviors and capabilities of elderly individuals in kitchen environments.

This study provides important insights into the postural demands placed on older adults when retrieving items from both upper and base kitchen cabinets; however, certain limitations must be acknowledged. Although the study considered group-level differences, specifically between men and women, and their influence on joint flexion angles, individual differences within these groups, particularly height, were not fully accounted for. For tasks involving upper cabinets, the results indicated that women exhibited greater peak shoulder flexion angles than men due to their generally shorter stature. However, the analysis did not adjust for individual height variations within each gender group, meaning that taller women may have experienced less shoulder strain compared to shorter women, and the same could apply to men. This limitation may have introduced variability in postural data, especially for tasks that required reaching up to retrieve items from higher cabinets. In the case of base cabinets, where participants were required to bend to retrieve items near the ground, individual height differences appeared to have a smaller effect, as the task uniformly required participants to bend at similar angles. However, we did observe that the flexion angles of the hips and knees were generally greater in women than in men, which we attributed to differences in habitual movement patterns rather than height differences. While group-level patterns were captured, flexibility, mobility, and other individual physical characteristics that were not measured in this study may have influenced these findings and contributed to variations in postural strain.

Although the study focused on an elderly population, and we analyzed how group-level differences, such as gender, affected postural demands, a more detailed exploration of individual-level differences beyond gender, such as specific height, body proportions, and flexibility, would enhance the understanding of how these factors interact with task demands. Future research should adopt a more granular approach, incorporating these individual variables to provide a personalized understanding of ergonomic risks and further refine design recommendations for age-friendly kitchens that accommodate a broader range of physical characteristics. The lack of adjustment for individual height differences, particularly for tasks involving upper cabinets, may affect the generalizability of the findings. Future studies should seek to address these individual variations by incorporating adjustable cabinet heights or treating height as a continuous variable within the analysis to yield more precise ergonomic insights. Moreover, this study primarily recruited self-care elderly individuals with good functional abilities, and did not involve participants who required external support during the tasks. However, for elderly individuals with reduced functional abilities, it is common to use assistive devices such as holding onto cabinets or using walkers to complete similar tasks. This factor was not considered in the current study. Future research should focus on elderly individuals with lower functional capacity and explore the impact of such assistive devices on posture and physical strain, in order to provide broader insights for age-friendly design.

All posture estimation algorithms have inherent errors. For the OpenPose algorithm used in this study, these errors may stem from image resolution, lighting conditions, camera angles, and limitations of the algorithm itself. Image resolution and lighting conditions can reduce the accuracy of joint point detection, especially in cases of insufficient lighting or heavy shadows. Variations in camera angles can also affect the estimation of joint positions. To ensure the reliability of our results, we paid particular attention to maintaining consistent camera angles and good lighting conditions in the experimental environment, to minimize the impact of these factors on the accuracy of posture estimation. Furthermore, posture estimation errors can lead to inaccurate calculations of joint angles, thereby affecting the evaluation results of posture load. To mitigate these effects, we adopted multiple measurements and averaged the results in our experimental design, along with detailed data verification. Despite these efforts, algorithm errors remain inevitable. Future research can improve the accuracy of posture estimation by introducing more advanced algorithms or combining other sensors, such as depth cameras or IMU sensors. Additionally, developing error correction algorithms and data post-processing techniques will also help reduce the impact of errors on the results.

Furthermore, the limitations of the equipment used in this study contribute to certain constraints. We solely utilized a Logitech C930C camera to capture 2D keypoint data. While this method is convenient, it lacks the ability to capture depth information in the three-dimensional space, potentially reducing the accuracy of assessments in certain complex movements. Additionally, the scope of behavior assessment is not broad enough. This study primarily focuses on the assessment of object retrieval movements, failing to comprehensively cover other common actions in the kitchen performed by the elderly, such as cooking and cleaning. Therefore, future research should incorporate 3D depth sensors, such as the Kinect, to obtain more precise three-dimensional keypoint data, thereby improving the accuracy of motion assessments. Diversifying the aspects of behavior assessment and expanding the range of evaluated behaviors to include more daily kitchen activities is also recommended. This would provide a comprehensive evaluation of the overall operational abilities and postural load of the elderly. Through further research, we aim to provide more comprehensive and accurate theoretical support for designing elderly-friendly kitchens, ultimately enhancing the quality of life for the elderly in their home environments.

## Conclusion

This study evaluates and analyses the behaviour of elderly individuals in retrieving items from cupboards by utilising questionnaires, field research, user interviews, and other methods. The research incorporates OpenPose technology and the REBA evaluation method to establish a framework for assessing the risk of Work-Related Musculoskeletal Disorders (WMSDs) during manual handling activities. The experiment involved forty-two self-sufficient elderly participants in operation behaviour analysis, with an equal male-to-female ratio. Data were recorded using a Logitech C930C camera, and the OpenPose algorithm was used to measure the relative positions of key points and calculate the corresponding angle values. The analysis was conducted using Origin software. The findings indicate that women exhibit a greater peak flexion angle in their right shoulder compared to men when retrieving items from upper cabinets, which can be attributed to differences in height between genders. Furthermore, taller subjects demonstrate a reduced peak shoulder buckling angle during upper cabinet operations. In contrast, when retrieving items from lower cabinets, women display a significantly larger peak flexion angle in both their left and right hips compared to men, with this discrepancy attributed to gender-specific living habits rather than height variation. Subsequently, motion data for each body part was determined from the collected data, and the action risk levels at different stages were assessed by combining with the REBA evaluation method. A comprehensive analysis of 42 video recordings yielded gradient data on the comfort levels of the subjects’ necks, trunks, and knees, with further statistical analysis conducted using Origin software indicating significant effects on neck, trunk, and knee comfort levels (*P* < 0.001). Subjects were able to differentiate between ‘easy’, ‘moderate’, and ‘strenuous’ tasks, thereby validating the collected data and establishing a comfort gradient model for reaching and bending behaviour. Given the common occurrence of skeletal joint degeneration and osteoporosis among elderly individuals, retrieving items in the kitchen becomes increasingly challenging with age. By devising an assessment framework based on human posture and muscle load for assessing elderly individuals’ retrieval capabilities, targeted improvement recommendations can be proposed to alleviate the operational burden on the elderly. This research contributes to enhancing the design of cabinets, thereby enhancing the comfort of elderly individuals during item retrieval and reducing the risks associated with their behaviours and postures. Consequently, this study serves to enhance the quality of life for elderly individuals at home and promotes the development of age-friendly designs for healthy aging.

## Supplementary Information

Supplementary Information.

## Data Availability

The datasets used and/or analyzed during the current study are available from the corresponding author upon reasonable request.
