# Supplementary material for: Evaluating functional ability in older adults’ object retrieval behavior from kitchen furniture using OpenPose and REBA
Source: Sci Rep. 2024 Oct 26;14:25560. doi: 10.1038/s41598-024-75470-6 (PMC11513087; doi:10.1038/s41598-024-75470-6)
Supplement: Supplementary file 1 — Supplementary Information. [file 41598_2024_75470_MOESM1_ESM.docx]

**Appendix A**

Questionnaire on Elderly People's Kitchen Usage

Dear Participant, thank you for taking the time to complete this questionnaire. The purpose of this survey is twofold: First, to understand and analyze the experiences of elderly users with kitchen cabinets. Second, to assess your awareness and expectations regarding age-friendly kitchen cabinets. This survey does not involve any personal information. Please respond based on your actual situation and genuine experiences. This questionnaire is intended solely for academic research and will not be used for any other purposes. Your personal information will remain confidential. We sincerely appreciate your participation.

1. What is your age? [Single Choice] *

○ 60-64 years old

○ 65-69 years old

○ 70-74 years old

○ 75-79 years old

○ 80 years old and above

2. What is your gender? [Single Choice] *

○ Male

○ Female

3. What is your current living arrangement? [Single Choice] *

○ Living with children

○ Living with spouse

○ Living with spouse and children

○ Living alone

○ Other _________________ *

4. What is the openness of your kitchen? [Single Choice] *

○ Open kitchen, no partitions

○ Semi-open kitchen, using glass doors or windows as partitions

○ Closed kitchen, separated by walls and doors

○ Other _________________ *

5. What is the layout of your kitchen? [Single Choice] *

○ L-shaped layout

○ Straight line layout

○ U-shaped layout

○ Parallel layout

○ Island layout

○ Other _________________ *

6. How frequently do you cook at home per week? [Single Choice] *

○ 1-2 days/week

○ 3-5 days/week

○ More than 5 days/week

○ Other _________________ *

7. How many people usually use the kitchen at the same time? [Single Choice] *

○ 1 person

○ 2 people

○ 3 or more people

8. Under what circumstances do multiple people use the kitchen simultaneously? [Single Choice] *

○ When there are guests at home, preparing meals

○ When retrieving items from upper cabinets

○ Rarely happens

○ Other _________________ *

9. Please rate the difficulty of washing vegetables in the kitchen: [Single Choice] *

Very easy ○ 1 ○ 2 ○ 3 ○ 4 ○ 5 ○ 6 ○ 7 Very difficult

10. Please rate the difficulty of cutting vegetables in the kitchen: [Single Choice] *

Very easy ○ 1 ○ 2 ○ 3 ○ 4 ○ 5 ○ 6 ○ 7 Very difficult

11. Please rate the difficulty of stir-frying in the kitchen: [Single Choice] *

Very easy ○ 1 ○ 2 ○ 3 ○ 4 ○ 5 ○ 6 ○ 7 Very difficult

12. Please rate the difficulty of cleaning in the kitchen: [Single Choice] *

Very easy ○ 1 ○ 2 ○ 3 ○ 4 ○ 5 ○ 6 ○ 7 Very difficult

13. Please rate the difficulty of retrieving items from upper cabinets in the kitchen: [Single Choice] *

Very easy ○ 1 ○ 2 ○ 3 ○ 4 ○ 5 ○ 6 ○ 7 Very difficult

14. Please rate the difficulty of retrieving items from lower cabinets in the kitchen: [Single Choice] *

Very easy ○ 1 ○ 2 ○ 3 ○ 4 ○ 5 ○ 6 ○ 7 Very difficult

15. Which parts of your body experience discomfort during kitchen tasks? [Multiple Choice] *

□ Neck

□ Shoulders

□ Waist

□ Arms

□ Wrists

□ Legs

□ Other _________________ *

16. Which activities cause discomfort in your neck? [Multiple Choice] *

□ Washing vegetables

□ Cutting vegetables

□ Cooking

□ Cleaning

□ Retrieving items from upper cabinets

□ Retrieving items from lower cabinets

□ Other _________________ *

Relies on (Question 15) Option 1

17. Which activities cause discomfort in your shoulders? [Multiple Choice] *

□ Washing vegetables

□ Cutting vegetables

□ Cooking

□ Cleaning

□ Retrieving items from upper cabinets

□ Retrieving items from lower cabinets

□ Other _________________ *

Relies on (Question 15) Option 2

18. Which activities cause discomfort in your waist? [Multiple Choice] *

□ Washing vegetables

□ Cutting vegetables

□ Cooking

□ Cleaning

□ Retrieving items from upper cabinets

□ Retrieving items from lower cabinets

□ Other _________________ *

Relies on (Question 15) Option 3

19. Which activities cause discomfort in your arms? [Multiple Choice] *

□ Washing vegetables

□ Cutting vegetables

□ Cooking

□ Cleaning

□ Retrieving items from upper cabinets

□ Retrieving items from lower cabinets

□ Other _________________ *

Relies on (Question 15) Option 4

20. Which activities cause discomfort in your legs? [Multiple Choice] *

□ Washing vegetables

□ Cutting vegetables

□ Cooking

□ Cleaning

□ Retrieving items from upper cabinets

□ Retrieving items from lower cabinets

□ Other _________________ *

Relies on (Question 15) Option 6

21. Which activities cause discomfort in your wrists? [Multiple Choice] *

□ Washing vegetables

□ Cutting vegetables

□ Cooking

□ Cleaning

□ Retrieving items from upper cabinets

□ Retrieving items from lower cabinets

□ Other _________________ *

Relies on (Question 15) Option 5

22. What inconveniences do you encounter while washing vegetables? [Multiple Choice] *

□ Water splashes onto the floor

□ No place to put the washed vegetables

□ The sink countertop is too low, causing waist fatigue

□ Other _________________ *

23. What inconveniences do you encounter while preparing ingredients? [Multiple Choice] *

□ Cannot see the knife clearly

□ Cannot hold the knife handle firmly

□ Bending over for too long, causing waist fatigue

□ Preparation area is small, nowhere to place the prepared dishes

□ Other _________________ *

24. What inconveniences do you encounter while cooking? [Multiple Choice] *

□ Slow movements

□ Forgetfulness

□ Other _________________ *

25. What inconveniences do you encounter while cleaning? [Multiple Choice] *

□ Water splashes onto the floor

□ The sink countertop is too low, causing waist fatigue

□ Other _________________ *

26. What inconveniences do you encounter while organizing the kitchen? [Multiple Choice] *

□ Cannot reach the upper cabinet items

□ Difficult to squat down to get the lower cabinet items

□ Small storage space

□ Inconvenient garbage disposal

□ Other _________________ *

27. How do age-related physical changes affect your cooking? [Fill-in-the-blank]

28. Do you ever forget to turn off the stove or electric appliances while cooking? [Single Choice] *

○ Never

○ Occasionally

○ A few times

○ Often

29. Would you like a timer reminder function? [Single Choice] *

○ Yes

○ No

○ Indifferent

30. What type of timer reminder would you prefer? [Single Choice] *

○ Flashing light

○ Sound reminder

○ Sound and light reminder

○ Other _________________ *

Relies on (Question 29) Option 1,3

31. What additional features would you like in the kitchen? [Multiple Choice] *

□ Safety monitoring – danger alert

□ Adjustable height cabinets

□ Adjustable height countertops

□ Localized lighting

□ Dietary recommendations

□ Double sink

□ One-touch emergency call

□ Other _________________ *

32. Can you accept a countertop with varying heights? [Single Choice] *

○ Yes

○ Consider after trying

○ Absolutely not
